# The Role of Methyl Donors of the Methionine Cycle in Gastrointestinal Infection and Inflammation

**DOI:** 10.3390/healthcare10010061

**Published:** 2021-12-29

**Authors:** Joseph A. Vaccaro, Saleh A. Naser

**Affiliations:** Division of Molecular Microbiology, Burnett School of Biomedical Science, College of Medicine, University of Central Florida, Orlando, FL 32816, USA; javaccaro97@Knights.ucf.edu

**Keywords:** S-adenosyl methionine, folate, vitamin B12, methylation, inflammation, methionine

## Abstract

Vitamin deficiency is well known to contribute to disease development in both humans and other animals. Nonetheless, truly understanding the role of vitamins in human biology requires more than identifying their deficiencies. Discerning the mechanisms by which vitamins participate in health is necessary to assess risk factors, diagnostics, and treatment options for deficiency in a clinical setting. For researchers, the absence of a vitamin may be used as a tool to understand the importance of the metabolic pathways in which it participates. This review aims to explore the current understanding of the complex relationship between the methyl donating vitamins folate and cobalamin (B12), the universal methyl donor S-adenosyl-L-methionine (SAM), and inflammatory processes in human disease. First, it outlines the process of single-carbon metabolism in the generation of first methionine and subsequently SAM. Following this, established relationships between folate, B12, and SAM in varying bodily tissues are discussed, with special attention given to their effects on gut inflammation.

## 1. Introduction

The methionine cycle plays a crucial role not merely in the regulation of the essential methionine but also in the generation of S-adenosyl methionine (SAM), a ubiquitous cofactor necessary for methyltransferase reactions. Folate (vitamin B9) and cobalamin (vitamin B12) are cofactors necessary for successful methionine regeneration from homocysteine, a vascular risk factor, and for maintaining an abundant level of intracellular SAM. Deficiency in SAM or its precursors leads to dysregulation of crucial methylation and cellular dysfunction. In this review, we discuss the metabolic pathways responsible for generating methionine and SAM, and the consequences of SAM deficiency in gastrointestinal tissue. We also discuss the effects of folate and B12 deficiency in correlation with SAM depletion in clinical studies, animal models, and cell culture systems. The observations collated in this review highlight the complex role of methionine and SAM in human physiology and disease.

## 2. Folate, B12, and the Methionine Cycle

### 2.1. Folate and B12

Folate, also known as vitamin B9, is a crucial vitamin in humans isolated from spinach in 1941. The name folate was derived from the Latin word for leaf, folium [1]. Folate supplementation soon showed similar effects as yeast and liver extracts in the prevention of megalocytic anemia [2]. Leafy vegetables and citrus fruits are high in natural folates, and the synthetic form of folate, folic acid, has been mandated as a grain supplement by the US Food and Drug Administration since 1998 [1,3,4]. Probiotic intestinal bacteria also have been known to synthesize folate and secrete it into the environment [5]. In mouse models, this phenomenon has been shown to alleviate colitis [6]. Deficiency of folate leads to impaired red blood cell generation, resulting in macrocytic anemia; during pregnancy, increased folate consumption is required to avoid neural tube defects in the fetus [7,8]. Polymorphisms in genes related to folate metabolism such as MTHFR and MTHFD (encoding methylenetetrahydrofolate reductase and dehydrogenase, respectively) have been correlated with complications and spontaneous abortion during pregnancy, though sometimes only when there are compound mutations [9,10]. For some patients, a varied diet is insufficient to avoid folate deficiency: chronic malabsorption or improper folate storage can be caused by alcoholism, inflammatory bowel disease, celiac disease, and tropical sprue [11,12,13,14,15]. Folate comprises a pteridine ring, para-aminobenzoic acid, and at least one glutamic acid (Glu) residue. Glu residue count is highly variable, and the synthetic vitamin folic acid contains only one [16,17]. In the case of polyglutamic folates, the additional Glu residues are removed by the intestinal mucosa prior to release into circulation [18].

**Figure 1:**
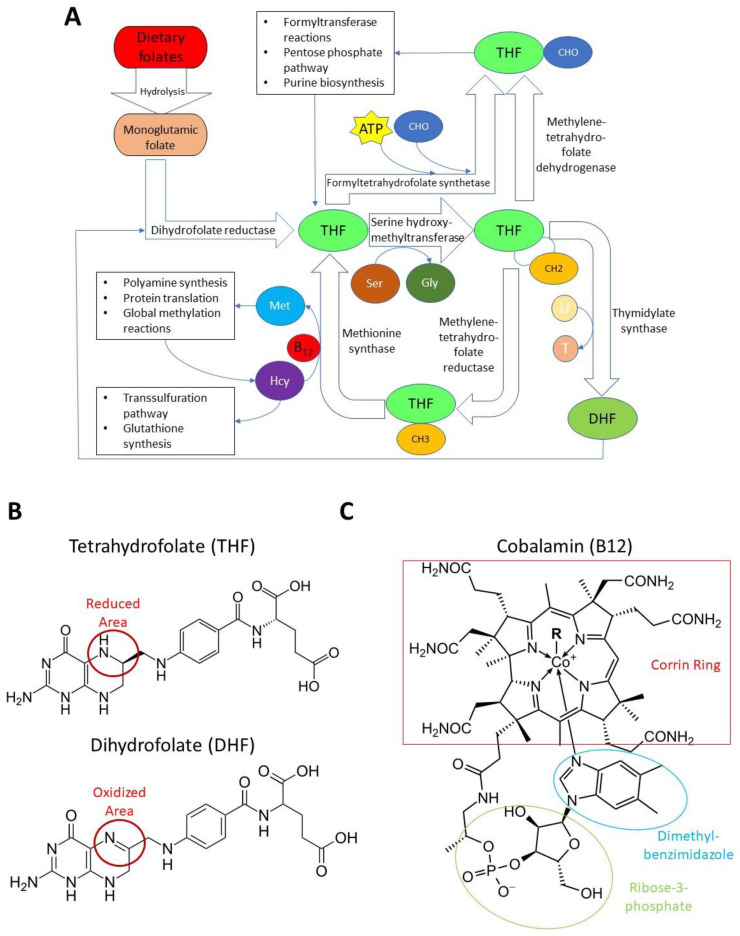
Outline of folate metabolism. Enzymatic processing of dietary folate to metabolically active cofactors and subsequent participation in single-carbon metabolism (**A**). Molecular structure of dihydrofolate DHF and tetrahydrofolate (**B**) as well as B12 (**C**). CHO: free formaldehyde or a formyl group. CH2: methylene group. CH3: methyl group. Met: methionine. Hcy: homocysteine. Ser: serine. Gly: glycine.

Both natural folates and synthetic folic acid undergo similar metabolic processing before their function as a coenzyme is realized—they are first absorbed by the upper small intestine by a carrier protein in the epithelium [19]. Absorbed folates are converted to dihydrofolate (DHF) and tetrahydrofolate (THF) by the enzyme dihydrofolate reductase (DHFR) [20]. Upon conversion to THF, folate can participate as a cofactor in single-carbon metabolism. THF serves as a transient carrier of methyl groups via a two-enzyme process. Serine hydroxymethyltrasferase (SHMT) catalyzes the conversion of THF and serine to glycine and 5,10-methylenetetrehydrofolate, using vitamin B6 as a cofactor [21]. Methylenetetrahydrofolate reductase (MTHFR) then converts the latter product into L-5-methyltetrahydrofolate [22]. However, this is not the only method of processing folate; 5,10-methylenetetrahydrofolate can be used by thymidylate synthase (TS) to convert deoxyuridine monophosphate (dU) to deoxythymidine monophosphate (dT), yielding DHF in the process as reviewed by Wilson and Mertes [23]. In addition to this metabolic product, formyltetrahydrofolate synthetase (FTHFS) fuses a free formic acid with THF to generate 10-formyltetrahydrofolate, an essential precursor in purine biosynthesis [24,25]. Alternatively, 5,10-methylenetetrahydrofolate can be processed via methylenetetrahydrofolate dehydrogenase (MTHFD) to generate the same metabolite [26]. For this review, the term ‘folate’ will refer to folates before their metabolism into a coenzyme (as they exist both naturally and synthetically in the diet) and their coenzyme forms. A schematic representation of folate metabolism is shown in Figure 1.

Vitamin B12 consists of a corrin ring with a central cobalt molecule; attached to the cobalt atom from above is a variable ligand distinguishing bioactive and dietary forms of the vitamin, while below is a ribose-3-phosphate-dimethylbenzimidazole ligand [27,28,29]. Some species in the human intestinal microbiota have been found to synthesize B12, though it is not believed that gut bacteria serve as a significant source of B12 in humans [30]. Like folate, it requires biochemical modification before it can participate in metabolism. Human biochemistry makes use of adenosylcobalamin and methylcobalamin, which are generated from dietary hydroxocobalamin and cyanocobalamin. Unlike folate, only two reactions in normal metabolism require vitamin B12 as a cofactor—the conversion of methylmalonyl-CoA to succinyl-CoA in the mitochondrion and the conversion of homocysteine to methionine in the cytoplasm. A more thorough review has been conducted by Allen and colleagues [31]. Despite this limited biochemical utility, B12 is essential, and deficiency results in pernicious anemia due to impaired red blood cell development [32]. This effect is sometimes masked by folate’s beneficial effect on erythropoiesis and can lead to misdiagnosis—a dangerous situation, as prolonged B12 deficiency also has deleterious effects on myelination and nervous system development [33,34,35]. Infants are particularly susceptible to neurological degeneration from B12 deficiency, and failure to restore normal levels can result in severe damage [36,37,38].

The metabolism of cobalamin, known as vitamin B12, is thoroughly intertwined with folate. B12 intestinal uptake is mediated by intrinsic factor (IF), a protein secreted by parietal cells in the stomach [39]. The IF-B12 complex is then taken up by the cubam receptor in the terminal ileum and transported through circulation via haptocorrin or transcobalamin protein carriers [40,41]. The metabolism of cobalamin, known as vitamin B12, is thoroughly intertwined with folate. Intrinsic factor (IF), a protein secreted by parietal cells in the stomach, mediates B12 intestinal uptake [39]. The IF-B12 complex is then taken up by the cubam receptor in the terminal ileum, released into serum by MRP1, and transported through circulation via haptocorrin or transcobalamin protein carriers [40,41,42]. Most cobalamin in serum is bound to haptocorrin; however, most cells cannot absorb the haptocorrin-B12 complex [43]. Instead, the ubiquitously expressed transcobalamin receptor (TCblR) mediates uptake of the transcobalamin-B12 complex into the cell via endocytosis [44,45]. Upon lysosomal degradation, the receptor is destroyed, and B12 is released; the proteins ABCD4 and LMBD1 are necessary for translocation across the lysosomal membrane [46,47]. MMACHC, also called CblC, receives the translocated B12 and catalyzes the removal of alkyl or cyanide ligands [48,49]. It coordinates with MMADHC, alternatively named CblD, an enzyme that facilitates cob(II)alamin oxidation to aquocobalamin [50]. The CblC/CblD complex interacts with methionine synthase (MS) and methionine synthase reductase (MSR) to ensure efficient cofactor delivery to its associated enzyme [51]. In addition, the enzyme methylmalonyl-CoA mutase in mitochondria requires adenosylcobalamin to function; however, the mechanism by which mitochondria take up cytosolic B12 remains unclear. One study in *C. elegans* suggests that an ABCG family protein may mediate membrane transport, but no mitochondrial membrane proteins have been identified in humans so far [52].

Any malfunction in this multi-factorial and complex process could lead to interruption in B12 uptake, causing deficiencies and ultimately an increased risk of developing disease. For example, autoantibodies against parietal cells or IF mutation may result in reduced IF secretion, which leads to B12 malabsorption and deficiency [53,54]. Similarly, damage to the ileum due to surgery or chronic inflammation, as seen in Crohn’s disease (CD), causes impairment in B12 uptake and increases the risk of developing additional symptoms and complications [55,56]. Metformin use in type 2 diabetes mellitus has also been correlated with decreased serum B12; however, the mechanism underlying this phenomenon is unclear [57,58]. Impaired intracellular B12 metabolism may likewise lead to metabolic dysfunction [59]. Accumulation of unabsorbed B12 causes bacterial overgrowth and inflammation [60]. Vegetarians, mainly vegans, are also susceptible to vitamin B12 deficiency due to a different cause—vitamin B12 is primarily found in animal products and is poorly represented in plants [61,62,63]. As such, nutritional supplements are sometimes indicated in this patient group; a more comprehensive review of B12 deficiencies in differing vegetarian diets has been performed by Pawlak and colleagues [64].

Folate and B12 belong to a class of vitamins known as methyl donors, a descriptor they share with choline and betaine, as summarized by Zeisel [65]. Methyl donors are so named because of their importance in single carbon metabolism, by which methyl groups are transferred from sources such as serine, glycine, and choline to a variety of other compounds, including proteins, RNA, DNA, and intermediate metabolites. The targets for methylation have been extensively reviewed elsewhere [66,67]. This process is accomplished via the folate and methionine cycles. L-5-methyltetrahydrofolate donates the methyl group attached to its 5′ carbon to B12 and subsequently homocysteine, converting it into methionine in a reaction catalyzed by methionine synthase (MS). This reaction yields tetrahydrofolate, which can participate in formyl and methyl group metabolism as described previously. Folate and B12 insufficiency leads to impeded methionine regeneration [68]. Methionine synthase’s continued function depends on the availability of methionine synthase reductase, an associated protein that reduces the nonfunctional Cb(II) ion of B12 to Cb (I), ensuring continuous cofactor function [69]. The importance of folate in fetal neural tube development has already been mentioned, but general methyl donor depletion in early life has been found to alter both long-term neurological changes in mice and intestinal development in rats [70,71,72]. When these findings are combined with research on the necessity of methyl donors for developing B cells, a picture of cell growth and differentiation emerges, highly dependent on methyl donor availability and methionine metabolism [73].

### 2.2. S-Adenosyl Methionine

S-adenosyl methionine is a modified form of the essential amino acid methionine, where an adenosyl group is covalently connected to the sulfur to generate a sulfonium ion. It serves as a universal methyl donor for a class of enzymes known as methyltransferases (MTases), which catalyze the transfer of methyl groups to biomolecules such as DNA, RNA, protein, and other metabolites that require them, as reviewed previously [66,67]. SAM is synthesized from methionine and ATP via the enzyme methionine adenosyltransferase (MAT), also called S-adenosyl methionine synthase [74]. SAM is a crucial precursor to the anti-inflammatory polyamines spermidine and spermine [75]. Removal of the methyl group by MTases converts SAM to S-adenosyl homocysteine (SAH), subsequently degraded into adenosine and homocysteine by SAH hydrolase. This reaction can be inhibited by adenosine dialdehyde and similar compounds, small-molecule SAH analogs. These compounds have been used as indirect methyltransferase inhibitors by halting the cycle at this point, consequently impeding methionine regeneration and causing SAH buildup, as SAH is a methyltransferase inhibitor [76].

**Figure 2:**
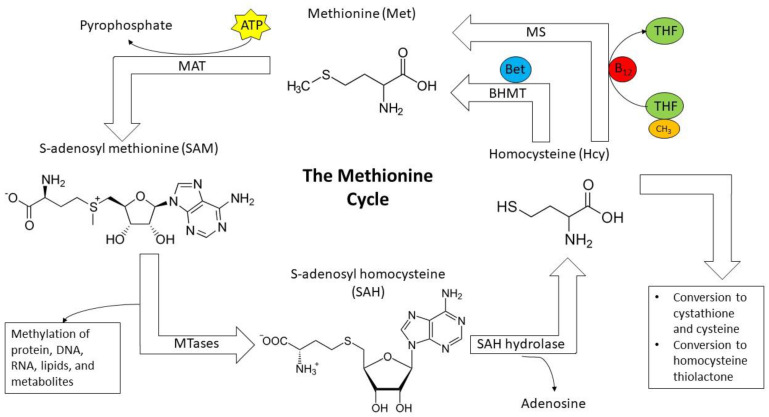
The methionine cycle. Methionine is converted to SAM by methionine adenosyltransferase (MAT) and subsequently used as a methyl donor in methyltransferase reactions. This yields SAH as a byproduct, hydrolyzed to homocysteine. Homocysteine is removed from the cycle by conversion to homocysteine thiolactone or through the trans-sulfuration pathway to cysteine. Alternatively, it is remethylated by one of two pathways: using methionine synthase with B12 as a cofactor and 5-methyltetrahydrofolate as a methyl donor or using betaine homocysteine methyltransferase and betaine as a methyl donor.

Following hydrolysis, homocysteine remains in the body as an intermediate metabolite and nonessential amino acid with several potential fates. It can be converted to homocysteine thiolactone with Met-tRNA synthetase and joined to proteins via oxidation with thiol groups [77]. Homocysteine can also be processed by cystathionine-β synthase to yield cystathionine, which is subsequently converted to cysteine via cystathionine-γ lyase using vitamin B6 as a cofactor. The process is referred to as the trans-sulfuration pathway, and it is crucial to the successful removal of SAH, a potent MTase inhibitor [78]. Alternatively, it can be remethylated to methionine via one of two pathways. Previously we mentioned vitamin B12’s cofactor activity in coordinating the removal of a methyl group from 5-methyltetrahydrofolate in converting homocysteine to methionine. In addition to this mechanism, betaine homocysteine methyltransferase (BHMT) can regenerate methionine from homocysteine by removing a methyl group from betaine, a derivative of the methyl donor and neurotransmitter choline [79]. This process is summarized in Figure 2. Interestingly, the enzymes in the pathway are also used for the metabolism of selenocysteine and selenomethionine, which are structurally similar to their sulfur-containing counterparts but far less common in the body [80,81].

Defects in methionine and SAM metabolism, either genetic or resulting from low levels of folate or B12, result in the buildup of one or more metabolites, associated with deleterious effects in the body. Of these, hyperhomocysteinemia, defined as excessively high serum homocysteine levels, has been associated with both global and tissue-specific inflammation, as well as deleterious effects on the vasculature and bone [82,83,84,85,86,87]. At another stage of the methionine cycle, hypermethionemia, or excessive levels of methionine, is also observed to alter cell proliferation; when artificially induced in culture, activated T cells divide more rapidly [88]. SAM can also serve as a precursor to purine nucleotides; as such, it can be administered as part of a combination therapy to patients with congenital abnormalities in purine biosynthesis to alleviate disease progression and symptoms [89].

## 3. Folate, B12, and SAM: Links to Tissue-Specific Inflammation

### 3.1. The Gastrointestinal Tract

Folate deficiency in IBS patients is neither surprising nor unprecedented. Studies as early as 1968 describe low serum folate in groups of Crohn’s patients, and a recent study found folate-associated metabolic pathways perturbed in CD and ulcerative colitis (UC) [90,91]. As such, folate metabolism has not gone unnoticed as a therapeutic target. In a pediatric IBD cohort dosed for one month with folate supplements, researchers found changes in micronuclei, nucleoplasmic bridge formation, and apoptosis in enterocytes and peripheral blood lymphocytes. These results were segregated by IBD typing—CD patients showed decreased signs of chromosomal damage with folate supplementation, while UC patients showed increased signs of it [92]. Folic acid supplementation reduced the incidence of side effects of methotrexate, an immunosuppressive drug and folate analog used to treat CD and rheumatoid arthritis [93,94]. A review of meta-analyses regarding environmental risk factors identified high folate levels as protective against the development of IBD—this suggests that the action of folate in the gut is not necessarily a reaction against the symptoms of IBD but may have a prophylactic effect [95]. Genetic factors controlling folate metabolism have been correlated with increased risk of IBD; the substitution of A2756 to G *MTR*, the gene encoding methionine synthase, is particularly notable for corresponding with IBD in a 2009 meta-analysis [96]. Examination of a mechanistic basis for this phenomenon is still not clearly understood.

Folate bioavailability has shown distinct effects on host–microflora interactions. Folate biosynthetic pathways are downregulated in intestinal bacteria during a CD relapse, an observation which pairs with the finding that folate-producing probiotic bacteria alleviate the inflammatory effects of chemically induced colitis in mice [6,97]. These phenomena are observed in other animal models as well; methyl donor-enriched diets in a mouse model of CD alter the expression of genes involved with colonization by adherent-invasive *E. coli*. The surface marker used by the pathogen to adhere to the epithelial lining was downregulated, as was calprotectin, an inflammatory marker, and IgA secretion. By contrast, antibacterial genes such as Lyz1 and Lyz2 were upregulated [98]. This study builds upon earlier findings in guinea pigs indicating that folate-deficient diets in early life sensitize animals to *Shigella* infection [99]. The connection between folate, B12, and the gut microbiome has been shown to work in both directions—*H. pylori* infection decreases gut uptake of folate and B12, leading to deficiency and pathogenic hyperhomocysteinemia [100].

These observations are not limited to folate; metabolic studies have shown perturbations in methionine metabolism and branched-chain amino acid oxidation in IBD, both pathways in which B12 is a crucial cofactor [91]. Furthermore, mutations in transcobalamin II, a protein responsible for B12 transport in serum, are associated with UC [101]. In a Swiss cohort of IBD that included CD, UC, and indeterminate colitis, B12 deficiency was associated with higher CD activity, stenosis, nephrolithiasis, and other complications [102]. Mouse models of colitis offer a complex picture. B12 deficiency leads to increased microbial dysbiosis in the gut flora and decreased enteric tissue damage. This effect may be caused by depletion of B12-dependent CD8+ T cells and NK cells in the gut, resulting in a minimized short-term inflammatory response to dysbiosis [103,104]. These results, however, have not been replicated in humans. Notably, B12 ligands were shown to have drastically different effects on inflammation in chemically induced murine colitis; cyanocobalamin appeared to worsen pathogenesis and inflammation, while methylcobalamin ameliorated them [105]. Conversely, methyl donor depletion in rats was shown to aggravate chemically induced colitis and lead to ER stress; it is uncertain precisely which compound’s absence was responsible for the effects observed [106].

Several studies have noted that perturbations in SAM availability and homocysteine recycling correlate or cause worsening IBD symptoms or complications. Independent of methyl donor levels, hyperhomocysteinemia has been correlated with osteoporosis in CD patients in both univariate and multivariate analyses. By contrast, folate deficiency was only correlated in univariate analysis [107]. In one study, vitamin B12 and SAM restriction independently led to endoplasmic reticulum stress mediated by SIRT1 reduction, which exacerbated colitis in rats [106]. In this species, induced colitis in conjunction with methyl donor deficiency correlates with hepatic inflammation and macrovesicular steatosis, where decreases in folate, B12, and the SAM/SAH ratio are closely correlated with inflammatory markers [108]. A murine model of colitis included SAM in a study of antioxidants as colitis treatment; SAM reduced serum amyloid A and TNF-α, improved reduced glutathione in circulation, and restored colonic length [109]. Unfortunately, there is a dearth of information assessing SAM’s effect on clinical IBD patients. While one study has noted an inverse relationship between SAM levels and IBD diagnosis or activity, clinical data are necessary to determine whether SAM supplementation ameliorates symptomatic disease and reduces complications [110]. Should clinical data confirm the prior findings, then mechanistic studies determining SAM’s effect will be warranted.

It is important not to overstate the significance of these findings; data are still mixed on the clinical effects of methyl donor deficiency, particularly in the long term. In the same Swiss study which found associations between B12 deficiency and CD complications, folate-normal patients had an increased occurrence of osteoporosis and fistula formation. Differences in treatment may explain this trend; patients treated with steroids, anti-TNF agents, or antibiotics had lower rates of folate deficiency but may have had more severe symptoms that necessitated such interventions [102]. A recent meta-analysis found no statistical decrease of serum folate levels in CD patients compared with healthy controls, though there was statistical significance in with IBD overall. The same analysis found that B12 concentrations were only significantly reduced in studies on Asian populations [13]. This finding is particularly noteworthy given a contradictory study which found folate and B12 deficiency specifically prevalent in CD patients compared with UC controls (22.2% and 15.6% prevalence compared with 4.3% and 2.8% prevalence, respectively), though indeterminate IBD was not studied [111]. Not all studies have correlated low serum folate or B12 with elevated homocysteine levels in CD, questioning the link between these nutrients and metabolic imbalance in IBD [112,113,114].

The above findings are indicative of a pathogenic role for folate and B12 deficiency in IBD. The precise interaction between these nutrients and IBD appears to segregate by IBD subtype, with some conditions worsening in UC but not CD or vice versa. Overall, worsening inflammation appears to be correlated with low folate and B12; however, future studies are warned to be wary of the applicability of animal models to human conditions. Furthermore, researchers investigating a mechanistic link between folate, B12, homocysteine, and SAM are strongly recommended to confirm the link between low methyl donors, high homocysteine, and low SAM in their patient sets.

### 3.2. Systemic Inflammation

Methyl donor availability, particularly folate and B12, alters various body-wide inflammatory conditions in chronic and acute disease models. In some models, a mechanism has been established; in others, only the characterization of the effect in specific tissues has been confirmed. In addition to its metabolic role as a cofactor, vitamin B12 protects against septic shock by scavenging nitrous oxide reactive oxygen species, which modulates both their inflammatory signaling effect and the damage incurred by the cell producing them. This antioxidative role is substantiated by the observation that supraphysiological doses of B12 have differential effects on NOS expression based on the organ in question in a model of toxic shock [115]. Furthermore, in a study analyzing the effects of vegetarian and omnivorous diets on B12 level and inflammatory status in diabetic patients, Lee et al. found that higher B12 levels in both dietary groupings were associated with lower IL-6 levels and increased catalase activity. However, the vegetarian group was more prone to deficiency [116].

Folate and B12 exert a significant effect on systemic inflammation via their canonical control of plasma homocysteine levels. Elevated homocysteine levels in serum have been found to mediate vascular inflammation by inducing cathepsin V expression and consequently the nuclear translocation of ERK1/2 [83]. This effect may be partially mediated by oxidative stress; treatment with selenium reverses the deleterious effects of homocysteine on endothelial cells and neuronal cells [117,118]. Sodium selenite has also been shown to reduce pathogenic clotting [119]. In addition, homocysteine stimulation results in NLRP3 inflammasome activation and resulting cellular stress via TXNIP—this mechanism contributes to homocysteine’s role as a risk factor in renal failure [120]. The failure to maintain low homocysteine results in increased oxidative stress, particularly in patients with chronic infection, sometimes with dangerous long-term effects; for example, sustained hyperhomocysteinemia can lead to pregnancy complications in hepatitis E patients [86].

Altogether, there is evidence that maintaining appropriate folate and B12 levels modulates systemic inflammation via an antioxidant effect and reduction of homocysteine, which is a risk factor in vascular disease.

### 3.3. Immune Cells

A substantial body of evidence discussing the role of methyl donors in inflammation continues to be gathered. Folate deficiency in RAW264.7 macrophages correlates with increased pro-inflammatory cytokine secretion in vitro [121]. Correlating with this observation is the finding that folate supplementation in cultured macrophages mitigates inflammation upon stimulation with LPS [122]. Notably, B12 deficiency has been shown to reduce phagocytosis and bactericidal activity in neutrophils collected from human peripheral blood, suggesting a role for B12 in leukocyte function [123]. Homocysteine has also been shown to augment inflammatory cytokine expression in vivo by affecting histone methylation in macrophages [124]. Aberrant B cell development has been observed in murine models of methyl donor depletion, with fewer B cells emerging from the pre-pro stage compared with controls [73].

Prior studies have noted relationships between SAM metabolism changes and differential function in various immune cell types, particularly T cells. CD8+ T cells have shown aberrant functionality in methionine-deficient cancer microenvironments, primarily manifesting as reduced cytokine production and increased apoptosis [125]. In CD4+ T cells, methionine adenosyltransferase is found to be inhibited by ethanol, sensitizing them to apoptosis and potentially explaining one factor of alcohol’s immunosuppressive properties. Survival after methionine adenosyltransferase inhibition is rescued by SAM supplementation [126]. This finding is of particular interest to patients with congenital hypermethionemia, typically due to mutations in methionine adenosyltransferase [127]. However, there are caveats with these observations, as other investigations have found that excessive methionine and methionine sulfoxide dosing has been correlated with M1 macrophage polarization and increased production of pro-inflammatory cytokines such as IL-6 and TNF-α [128,129]. This suggests that the effects of methionine and methionine sulfoxide excess may be a confounding factor in the investigation of SAM deficiency and hyperhomocysteinemia. On this point, mechanistic studies like those undertaken with hyperhomocysteinemia are warranted. Exploring in vivo effects based on observations in vitro requires substantial care; systemic inflammation can increase if T cell survival is biased towards pro-inflammatory subsets, and high methionine diets have been shown to predispose rats to Th17 cell polarization [88,130].

The findings cited above suggest an essential role for methyl donors and SAM in immune cell survival and function, modulating inflammation when there is an adequate amount of methyl donors. When this improves pathogen removal, the overall effect might be termed beneficial. However, in conditions involving an excessive immune response, increased survival of immune cells may lead to persistence of inflammation. Further study may clarify the utility of these ambiguous findings.

### 3.4. The Nervous System

While folate is well known for its importance in preventing neural tube defects in utero, its effects on the nervous system do not end after birth. For example, folic acid supplementation has been shown to mitigate inflammation in Alzheimer’s disease, decreasing serum TNF-α levels and slightly improving mental state in patients [131]. Folate’s importance to the nervous system is further implied by the association of MTHFR polymorphisms with a predisposition to migraine, which disappeared upon folate supplementation in a clinical trial by Di Rosa et al. [132]. Echoing these findings are associations between MTHFR, MTR, MTHFD1, and oxidative stress in patients with some types of neurodegenerative disease [133].

B12 has been implicated in neuroprotection in various models of disease. Postmortem sampling of prefrontal human cortexes indicated that B12 status was decreased with age. The same study simultaneously found decreased methylcobalamin levels and methionine synthase activity in the brains of autistic patients [134]. A clinical trial of methylcobalamin supplementation showed moderate effects against diabetic neuropathy [135]. Neuroprotective effects of B12 were also found in rat models of both epilepsy and bacterial meningitis, where B12 levels reduced oxidative stress, inflammatory cytokine expression, and hippocampal damage [136,137]. In mice with methionine-high and B6, folate, and B12-low diets, microhemorrhages and amyloid-plaque buildup was observed in neural tissue, corresponding with increased inflammatory cytokine expression, emphasizing the protective effect of methyl donors and the dangerous impact of homocysteine [138,139]. B complex supplementation was shown to have a beneficial effect on peripheral nerve repair; B vitamin injections reduced inflammatory cytokine expression, polarized macrophages towards an M2 phenotype, and induced an anti-inflammatory phenotype in Schwann cells following injury [140]. The fact that B12 is implicated in neuroprotection in both the central and peripheral aspects of the nervous system suggests that neural tissue has a particular dependence on vitamin B12 that is still being elucidated, with a specific interest in a putative role for injury prevention and repair.

Independent of folate and B12, SAM supplementation has shown striking effects on neuroinflammation and pathogenesis. Conversely, high levels of homocysteine are a risk factor for neurological disease [82]. SAM was reduced in the cerebrospinal fluid of Alzheimer’s disease (AD) patients, while homocysteine is notably elevated in AD patient plasma [84]. This reduction correlates with observations in 1995 of hypomethylation in the amyloid-β gene of an AD patient, in addition to observations of global methylation changes in the prefrontal cortexes of 12 AD patients compared with controls [141,142]. Notably, amyloid-β plaque buildup is reduced by SAM dosage in mouse models of AD [143]. Following ischemic stroke, a correlation between hyperhomocysteinemia and exaggerated STAT3 activation has been found in microglia; it also exacerbated long-term tissue damage [82,144]. At times, the distinction between methyl donor treatment and SAM treatment is not a well-defined one; vitamin B12 injection has shown efficacy at reducing hippocampal inflammation in a rat model of bacterial meningitis, and one of the mechanisms the authors identified as protective was increased availability of SAM, leading to increased methylation of CpG islands in the promotor of Ccl3 [136]. Interestingly, methionine restriction in helper T cells has been found to reduce neuroinflammation by preventing T-cell proliferation and differentiation into pro-inflammatory subsets in autoimmune diseases such as multiple sclerosis [88]. This observation highlights the continuing need for further study on global SAM effects in disease models, not merely tissue- or cell-specific studies.

From these findings, we conclude there is strong evidence for a beneficial role for folate, B12, and SAM in modulating neuroinflammation. These findings have been corroborated by investigators examining various types of neuroinflammation; the beneficent effects are promising for clinicians who are interested in prophylactic treatments for at-risk patients. We encourage further mechanistic studies on this topic to elucidate why the nervous system, in particular, has shown responsiveness to this approach, and which compound or treatment is most consistently beneficial.

### 3.5. The Liver

Liver disease has been correlated with decreased methyl donor availability in vivo and in vitro. The trend is especially evident in chronic hepatitis C infection. Egyptian hepatitis C and liver cirrhosis patients have been found to possess low serum folate and elevated plasma homocysteine; furthermore, folate levels positively corresponded with platelet count, indicating low thrombocytopenia [145]. In chronic hepatitis C patients, SAM treatment improved response to pegylated interferon and ribavirin therapy by altering the methylation status of STAT1 in cultured cells. This modification led to an enhanced downstream signaling effect, which enhanced the antiviral state of the treated cells [146]. Improved interferon and ribavirin therapy responses were also found with vitamin B12 supplementation, though the authors ascribe this finding to B12-mediated IRES inhibition independent of B12’s methyl donor activity [147]. The liver has been shown to have a particular sensitivity to methyl donor deficiency during induced colitis in one rat model, suggesting that these nutrients may have a protective role against hepatotoxic inflammation in other tissues [108].

Outside the strict paradigm of infection, SAM dosing has been found to reduce ethanol-induced apoptosis in primary hepatocytes. Interestingly, the authors found no indication that it altered JNK activity in the proapoptotic signaling cascade and have postulated that an antioxidant effect is responsible for the phenomenon [148]. The importance of SAM in hepatocyte survival in response to inflammatory oxidative stress was highlighted in a murine model of hepatitis, where SAM depletion led to liver failure and death [149].

In total, the literature evidence suggests a beneficial role for SAM in the resolution of infection and the modulation of apoptosis in the liver. However, the findings are far from conclusive and only examine liver health from the paradigm of infection.

### 3.6. Other Tissues

Psoriasis, a chronic inflammatory condition primarily mediated by T cells, has been shown to correlate with hyperhomocysteinemia, and folic acid derivatives or topical B12 treatment provided mild attenuation of inflammation in initial trials. Clinical trial patients found a B12 containing cream superior to a standard hydrating cream for alleviating symptoms [150,151,152]. Atopic dermatitis patients also found improved relief of symptoms using B12 cream compared to the unmodified control [153,154]. However, these effects are nutrient-specific; we could not find studies demonstrating folate as an effective intervention alone, though some utility has been noted for folate supplementation in conjunction with methotrexate treatment for psoriasis [155]. By contrast, vitiligo, a skin condition characterized by loss of melanocytes and skin pigmentation, was reduced or halted in a Swedish clinical trial that administered folic acid and intramuscular B12 [156].

Some studies have suggested a beneficial role for methyl donor supplementation in respiratory disease. Supplementation of cystic fibrosis patients with 5-methyltetrahydrofolate and vitamin B12 reduced red blood cell oxidative stress, even in patients who were folate and B12-normal [157]. Beneficent effects of methyl donors on oxidative stress were echoed in a murine model of chronic asthma, where oxidative stress, tissue remodeling, and Th2 cytokine production were all ameliorated by SAM treatment [158]. Methyl donating nutrients have also been found to have beneficial effects on the lungs. A Greek cohort of asthmatic girls was found to have an association between low serum folate and impeded lung function [159]. The observed antioxidative effects of betaine in the lungs in response to paraquat toxicity have been proposed to be mediated through liver-generated SAM [160]. Despite the beneficent effects previously described, SAM’s role in the airways remains complex; SAM abundance can be scavenged by opportunistic pathogens like *Pneumocystis* species, leading to pathogenesis [161].

**Figure 3:**
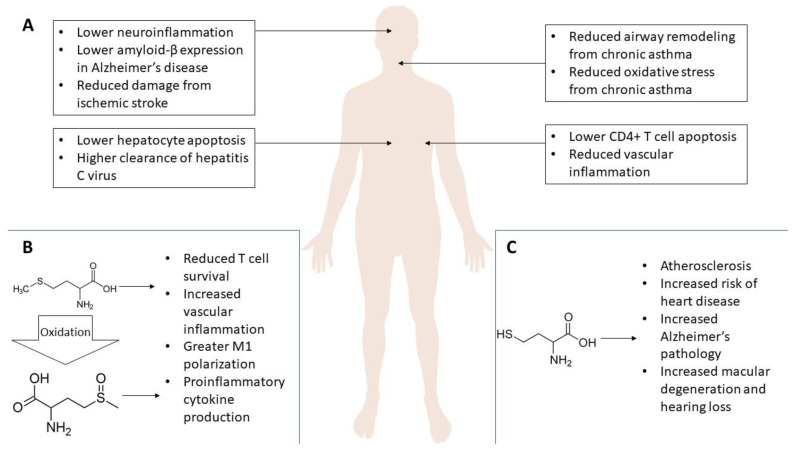
SAM and inflammation. Overview of the effects of SAM supplementation on the brain (**top left**), airways (**top right**), liver (**bottom left**), and spleen/vasculature (**bottom right**) (**A**). Possible model of the pathology of hypermethionemia, typically caused by defects in MAT preventing hepatic processing to SAM. Accumulation of methionine in the cell leads to incidental oxidation to methionine sulfoxide, necessitating enzymatic reversal (**B**). Summary of the effects of hyperhomocysteinemia on disease pathology (**C**).

**Figure 4:**
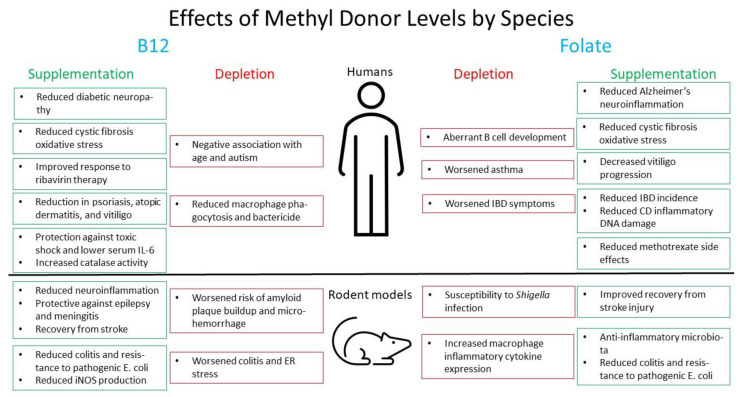
Overview of the effects of folate and B12 supplementation or depletion in humans and rodent models.

These results highlight the beneficial effects of methyl donors at epithelial tissues, which are consistently exposed to a broad category of microbes, some pathogenic. While more studies are indicated, the current results suggest a positive impact on immune regulation at these sites. It is too soon to conclude at this point that folate, B12, and SAM have an exclusive or even generally positive impact on these conditions, but there is more than enough justification to examine the effects in more detail. Figure 3 highlights the beneficent effects of SAM supplementation in humans. Figure 4 notes the summed effects of methyl donor depletion or supplementation in human and rodent models.

## 4. Conclusions

When one examines the effect of methyl donors on distinct tissue types, a distinct breakdown in effect by nutrient can be observed. In the gastrointestinal tract, folate, B12, and SAM show marked anti-inflammatory effects on IBD, though SAM lacks support from clinical patients. Folate and B12 further exert beneficent effects on systemic inflammatory markers by reducing serum homocysteine, with an additional antioxidative effect in vitamin B12. There is strong evidence that these vitamins also reduce neuroinflammation and promote neural tissue survival, though further mechanistic studies are warranted to determine how this effect is mediated. While present, direct evidence for SAM’s effect on systemic inflammation and neuroinflammation is comparatively lacking. Studies on immune cells also show anti-inflammatory effects upon folate and B12 dosage, with SAM enhancing cell survival. However, the pro-inflammatory effects of methionine excess make it challenging to determine whether SAM’s antiapoptotic effect will translate to reduced inflammation in vivo. Vitamin B12 and SAM bioavailability have been shown to affect hepatic cell survival and infection response, though folate data are comparatively lacking in this tissue. In epithelial cells and airways, aberrant immune responses are ameliorated by folate and B12, with SAM indicated as a likely mediator of their effects.

The findings collated in this review are striking not merely due to their variety, but the potency of their effects as well. Methionine metabolism and changes in S-adenosyl methionine levels are demonstrated to have wide-reaching and distinct effects on the same tissue type; similar phenomena are demonstrated for the methyl donating metabolites that control them. The complexity of the responses observed is a warning to those who might attempt therapies centered on methionine metabolism; when dealing with a metabolite crucial to many biological processes, it is necessary to tread carefully.

Despite these challenges, the state of the literature indicates that in some circumstances, folate, B12, and S-adenosyl methionine are capable of pleiotropic and powerful effects on inflammation. Given their ubiquity in the body and the continuing problem of chronic inflammatory disease, any dietary factor capable of complementing or replacing an anti-inflammatory pharmaceutical is especially advantageous. Naturally, the contradictory and complex findings necessitate further investigations to ensure SAM and methyl donor-based interventions are protective and do not exacerbate infection or inflammation. Building upon this, work in the future may enhance the capacity of physicians to engage the health of their patients in ways that correct nutritional imbalances, mitigate chronic conditions, and improve quality of life.

## Data Availability

Not applicable.

## Abbreviations

BHMT	Betaine homocysteine methyltransferase
Ccl3	Chemokine (C-C motif) ligand 3
CD	Crohn’s disease
CD4+	Cluster of differentiation 4-positive
CD8+	Cluster of differentiation 8-positive
DHF	Dihydrofolate
DHFR	Dihydrofolate reductase
ER	Endoplasmic reticulum
ERK1/2	Extracellular signal-regulated protein kinases 1 and 2
FTHFS	Formyltetrahydrofolate synthase
Gly	Glycine
Hcy	Homocysteine
IBD	Inflammatory bowel disease
IF	Intrinsic factor
IgA	Immunoglobulin A
IL-6	Interleukin 6
IRES	Internal ribosome entry site
JNK	C-Jun N-terminal kinase
LPS	Lipopolysaccharide
Lyz1/2	Lysozyme 1 and 2
MAT	Methionine adenosyltransferase
Met	Methionine
MS	Methionine synthase
MTases	Methyltransferases
MTHFD	Methylenetetrahydrofolate dehydrogenase
MTHFR	Methylenetetrahydrofolate reductase
NK cell	Natural killer cells
NLRP3	NLR family pyrin domain containing 3
NOS	Nitric oxide synthase
SAM	S-adenosyl-L-methionine
SAH	S-adenosyl homocysteine
Ser	Serine
SHMT	Serine hydroxymethyltransferase
STAT1/3	Signal transducer and activator of transcription 1 and 3
THF	Tetrahydrofolate
TNF	Tumor necrosis factor
TNXIP	Thioredoxin interacting protein
TS	Thymidylate synthase
UC	Ulcerative colitis
